# Perceived partial social integration, levels of distress and resilience, and COVID-19 vaccine rejection of Jewish and Arab citizens of Israel

**DOI:** 10.3389/fpubh.2022.1021015

**Published:** 2022-11-22

**Authors:** Yohanan Eshel, Shaul Kimhi, Hadas Marciano, Bruria Adini

**Affiliations:** ^1^Stress and Resilience Research Center, Tel-Hai College, University of Haifa, Haifa, Israel; ^2^ResWell - Multinational Resilience & Wellbeing Research Center, Tel Aviv University, Tel Aviv, Israel; ^3^Stress, and Resilience Research Center, Tel-Hai College, The Institute of Information Processing and Decision Making (IIPDM), University of Haifa, Haifa, Israel; ^4^Department of Emergency and Disaster Management, Sackler School of Public Health, Sackler Faculty of Medicine, ResWell - Multinational Resilience & Wellbeing Research Center, School of Public Health, Tel Aviv University, Tel Aviv, Israel

**Keywords:** COVID-19 pandemic, vaccine rejection, partial social integration, responses of majority and minority, distress, resilience

## Abstract

**Introduction:**

The present study examines the role of perceived partial social integration (PPSI) in determining the rejection of the COVID-19 vaccine of Jewish and Arab citizens of Israel.

**Methods:**

The research hypotheses are examined using a relatively large sample of the Israeli public, including 208 Arab and 600 Jewish adults, who have responded to an anonymous questionnaire pertaining, among other issues, to partial social integration and the individual level of vaccine uptake.

**Results:**

Higher levels of PPSI were found to be associated with higher levels of vaccine rejection, in both Jewish and Arab samples. The Arab minority group regards themselves as less socially integrated into the Israeli society and therefore rejects the COVID-19 vaccine to a greater extent than the majority group. The Arab respondents expressed a higher level of psychological distress and a lower level of resilience compared with the Jewish participants. The perceived partial social integration score significantly predicted the levels of distress and resilience of the Jewish but not the Arab sample.

**Discussion:**

The study indicates that increasing the vaccination rates depends more substantially on trust in the authorities than on leveraging greater pressure on individuals that reject the vaccine. Increased trust in the authorities and regarding oneself as an integral component of society are two vital conditions for vaccine acquiescence. Insufficient social integration is a major reason for vaccine rejection.

## Introduction

The present study examines the concept of perceived partial social integration (PPSI) as a psychological explanation for social behavior associated with the COVID-19 pandemic vaccination of members of the Jewish majority and the Arab minority population in Israel.

Social integration has been analyzed from two different perspectives. One viewpoint defines integration by behavioral indices: the number of social roles that an individual actively participates in, such as a parent, friend, student, or volunteer, as well as involvement in social interactions and different forms of human contact (1, 2). A second position emphasizes the experience of integration. Rather than counting social behaviors, it examines how individuals experience their place in society (3). According to this theoretical perspective, the essence of integration is a feeling of belonging, which affects perceived control and competence, and constitutes a major determinant of the individual's sense of wellbeing (4–6). A non-threatening social climate, characterized by comradeship and mutual support, is encouraged by open and fluid communication, whereas undesirable interactions negatively influence social integration and participation over time (7). According to this point of view integration is a two-way process, reflecting the interaction between the individual and the general society (3). In successful social integration, individuals regard themselves as authentic members of their society and trust that most members of this society accept them as integral parts of the general public. It has been found that in many cases such reciprocity is not guaranteed. PPSI is felt by those who do not regard themselves as full participants in their society (8).

The available research concentrates more often on the perspective of the prejudiced parties, indicating that declarations of social equality often involve implicit prejudices that take the form of the unconscious and/or automatic mental associations by which the members of one social group negatively evaluate another group (9, 10). The present study explores issues that have not been thoroughly studied by the available research on social integration: the behavioral and psychological impacts of PPSI on members of the Jewish majority and the Arab minority in Israel in the context of the COVID-19 pandemic. There is very little empirical research concerning the impact of PPSI on social behavior in general or on vaccination against the COVID-19 virus (11), and we are not aware of any study that compares its impact on the Arab and Jewish citizens of Israel. We believe that a sense of PPSI of disadvantaged groups, which has hardly been studied empirically, is liable to be found in any social community, including those who actively support the notion of social equality. A better understanding of the sense of PPSI and its implication for those who regard themselves as only partly integrated within their society is essential for comprehending the processes that impact social and political developments that are liable to threaten the integrity of any social organization, large or small. The notion of PPSI is likely, therefore, to contribute substantially to the research on social psychology and personality in the larger society, as well as in smaller communities and organizations.

It is assumed, first, that being vaccinated against the COVID-19 virus, which is a social behavior aimed at protecting the individual and the surrounding society in coping with the stressful conditions of this pandemic, will be negatively affected by a higher sense of PPSI of members of both the Jewish and the Arab communities, i.e., by perceived partial social acceptance and partial rejection. We expected that as Jews constitute the hegemony majority in Israel and the Arabs are a more marginalized national minority, the Arab respondents will regard themselves as less integrated in the Israeli society, will be characterized by a higher PPSI level, and will be vaccinated against the COVID-19 virus to a lesser degree than the Jewish respondents. Moreover, their higher level of distress will be expressed by higher levels of anxiety, depression, and sense of danger, accompanied by lower levels of optimism, societal resilience, and individual resilience, compared with the Jewish respondents. It has been argued that such a high level of psychological distress may further inhibit people from initiating or maintaining social contact with others (12–14).

Our second assumption predicted that a substantial difference between the Jewish and the Arab samples will be found in the association of their PPSI scores with expressions of distress and wellbeing. A higher sense of PPSI of the Jewish respondents will be associated with lower levels of optimism, societal resilience, and individual resilience as well as with higher levels of anxiety, depression, and sense of danger (as was found in a previous study of the current Jewish sample (11). Such expressions of distress of the Arab respondents will be dissociated from the sense of PPSI due to the psychological process of partial denial (15) that will not characterize the members of the Jewish majority.

### Perceived partial social integration (PPSI)

A considerable number of individuals share a perception of partial social integration (PPSI) and are not sure whether and to what extent they are accepted by their society. They keep wondering whether they are perceived as equal members of the society they belong to or feel that they are under-estimated by this society (16). Those who regard themselves as only partly socially integrated are likely to feel rejected and socially discriminated against (17). The extent and duration of such perceived partial social integration (PPSI) may result in varied negative social and personal consequences.

The concept of PPSI is an example of the process of social comparison which is an ever-present element of human social life. Looking to others for information directs people's thinking, feeling, and behavior, and provides them with criteria for assessing their relative social position. These comparisons help them consolidate a perception of how they are treated, covertly or overtly, by their society, feed into their attitude toward it, and continue to modulate their individual psychologies (18).

### Psychological variables associated with PPSI

#### Distress symptoms

Cénat et al. (19) have indicated that symptoms of anxiety and depression symptoms constitute the most common negative human reactions to threats and or disasters. Both their prevalence and association with each other are retained in the context of the COVID-19 pandemic (20). Perceived distress is negatively associated with wellbeing and resilience (21).

#### Sense of danger

Solomon and Prager (22) have assumed that a sense of danger is evoked by conditions of menace and disaster and is heightened by a concern for the wellbeing of dear ones. These feelings, like symptoms of stress, are negative indicators of individual coping (23).

#### Individual resilience (IR)

Luthar et al. (24) define individual resilience as “a dynamic process encompassing positive adaptation within the context of significant adversity” (p. 543). The American Psychological Society defines resilience as a process of “bouncing back” from difficult experiences and “adapting well in the face of adversity, trauma, tragedy, threats or significant sources of stress” (25). It has been found that strengthening individual resilience may help the adoption of positive coping styles, and promote mental health and psychological wellbeing (26, 27).

#### Societal (national) resilience (SR)

SR pertains to individuals' belief that the national authorities are competent and can be trusted to cope with adversities and to recover quickly after the threat was removed (28, 29). Societal resilience is negatively predicted by distrust in the authorities and distrust in the COVID-19 vaccine (30).

#### Optimism

Optimism is the expectation that good outcomes will occur. Optimists are more likely to choose problem-focused coping strategies when they are in situations they can control (31). Optimism reflects the extent to which people hold generalized favorable expectancies for their future. Higher levels of optimism are related to better subjective wellbeing in times of adversity or difficulty. Furthermore, optimism is linked to higher levels of engagement coping and lower levels of avoidance coping and is associated with taking proactive steps to protect one's health (32). Thus, it has been found that optimism is related to adaptive responses to the COVID-19 pandemic (33).

### Arab citizens of Israel

The Arab citizens of Israel constitute 21% of the country's population (34). Compared with the Jewish Israeli society the Arab society is less individualistic and more authoritarian, emphasizing connectivity and social relationships with meaningful others in one's social environment (35). Members of the Arab minority also differ from the Jewish majority in terms of language, religion, and other cultural factors (36), and are characterized, on average, by lower levels of education and economic conditions (37). Approximately 90% of the Arab minority reside in homogeneous Arab villages or cities, and about 10% of them reside in mixed Jewish and Arab cities (38). The civic identity of young Arabs in Israel is shaped, therefore, with limited or no interaction with the Jewish majority (39), and their national resilience is significantly lower than this resilience of the Israeli Jews (40).

The high level of stress among the Israeli Arab respondents reflects most probably several causes. First, they are part of a society transitioning from a traditional to a modern ways of living and thinking. An acculturation stress may arise from the need to learn new cultural rules and expectations, and the necessity to cope with an intrinsic conflict of wishing to retain important elements of the old culture while incorporating elements of the new one (41).

Furthermore, despite enjoying full citizenship status, the Arab minority still has a marginal status in the Israeli society and is subject to various forms of discrimination, as well as harsh living conditions (42–44). These stressful experiences may account for the severe violence that is evident within the Arab communities in recent years. Research shows that stressful conditions are likely to result in mental-health problems such as anxiety and depression, feelings of alienation, identity confusion, and heightened levels of psychosomatic symptoms (45, 46).

Israeli studies show that at the individual level the Arab population suffers a higher level of emotional distress and lower levels of interpersonal and transpersonal hope (47), compared with the Jewish majority, as well as from more psychological symptoms of distress (e.g., depression, somatization and anxiety) than their Jewish counterparts (37, 42). Arab citizens of Israel are likely therefore to regard themselves as less integrated into the Israeli society, more excluded by it, and only partly accepted by the majority group as equal citizens.

We assume that the PPSI scores of the Jewish sample will be associated with a higher level of distress and a lower level of individual resilience, whereas the PPSI scores of the Arab sample will not be significantly linked to their reports of psychological afflictions and wellbeing. Mariotti (48) and Yaribeygi et al. (49) argue that the impact of psychological stress depends on the individual perception of its controllability. A low level of perceived control over continuous stress is likely to damage one's adaptation and may cause mental illnesses such as depression and anxiety disorders. Effective regulation of negative emotions is required for adaptive coping with a sense of distress. One defense mechanism which can reduce the anxiety raised by extreme stress is denial, which is defined as an “automatic psychological process that protects the individual against anxiety and from awareness of internal or external stressors or changes” [(50), p. 765]. The more adaptive form of this defense mechanism is partial denial in which a high-risk situation is rated lower than less risky threats (51). Partial denial is a very common phenomenon in cases of illness. Chronic patients, who are well aware of their physical condition, may be partly reluctant to acknowledge health-related information and its effect on their lives (52–54). This partial denial of threats is an emotionally focused process, aimed at supporting individual adjustment to harmful and traumatic external events, which may sometimes be considered as adaptive behavior, supporting people's resilience (55).

Our previous study (11) shows that in the context of the COVID-19 pandemic, a lower level of social integration of Israeli Jews predicts a higher level of distress. As indicated above, the Arab citizens of Israel tend to be in a state of distress, and many of them regard their daily life as constantly distressing. They should be expected, therefore, to express higher levels of psychological distress and sense of danger compared with the Jewish sample, as well as a lower level of personal resilience. We assume that despite the distress of the low social integration level of the Arab respondents, the refusal of the Jewish majority to accept them as equal members of the Israeli society and their implications, they will employ a process of partial denial, and dissociate their experience of psychological distress from their PPSI.

The following hypotheses are investigated:

(1) The Arab respondents will score higher on PPSI, distress symptoms and sense of danger, and will score lower on individual resilience, societal resilience (SR), optimism and vaccination uptake, compared with the Jewish respondents.
(2) Higher PPSI scores of both groups will predict lower SR and lower level of vaccine uptake.
(3) Higher PPSI scores in the Jewish sample will positively predict depression and a sense of danger and will negatively predict optimism and individual resilience. Higher PPSI scores of the Arab sample will not significantly predict these indicators of distress and positive coping.

## Methods

### Data collection

The data were collected between October 8–12 in 2021, at the height of the fourth wave of the COVID-19 pandemic, *via* an internet panel company possessing a large database of residents from all demographic sectors and geographic locations of Israel. A stratified sampling method was employed, aligned with the data of the Israeli Central Bureau of Statistics, in an attempt to reach the varied groups of the Israeli populations (regarding gender, age and family income). The questionnaire was approved by the Ethics Committee of Tel Aviv University and all the participants expressed their informed consent.

### Data analysis

Path analyses, Amos Structural Equation Modeling, examined our hypotheses. The predictor variables were controlled for each other [IBM, SPSS, https://www.ibm.com/il-en/marketplace/structural-equation-modeling-sem; (56)]. Maximum likelihood estimates were employed and examined a saturated model, as we did not find any studies that supported an alternative model. It is important to note that in a saturated model, there is no need to examine a model fit as the default and the saturated model are the same (57).

### Participants

The present sample includes 600 Jewish and 208 Arab adults. Table 1 presents their demographic variables and shows that their ages range from 18 to 84 years. The Arab sample includes a higher percentage of the younger age group and a lower percentage of the elderly. 51% of the Jews are females and 49% are males, whereas 48% of the Arabs are males and 52% are females. The mean family income of the Jewish sample is significantly higher than the income of the Arab participants, and the political attitudes of the Jewish sample tend to be significantly more right-wing than those of the Arabs. No significant education level is found between the two groups. Israel is among the countries with the highest levels of vaccination for COVID-19, with 78% of those 12 years and older fully vaccinated (58).

**Table 1:** Demographic characteristics and analysis of variance (ANOVA) comparing mean scores of Jewish (*N* = 600) and Arab sample (*N* = 208).

**Variable**	**Sample**	**Distribution**			**M**	**SD**	**F**	**Cohen's d**
(scale)	Range	n	%				
Age	Jewish	18–30	178	30	42.13	16.33	12.49^***^	0.38
		31–40	124	21				
		41–50	124	21				
		51–60	84	14				
		61 and above	90	15				
	Arabs	18–30	74	36	37.70			
		31–40	51	24		1.31		
		41–50	43	21				
		51–60	28	13				
		61 and above	12	6				
Gender	Jews	Men	291	48	-	-	–	–
		Women	309	52	-	-		
	Arabs	Men	106	51	-	-		
		Women	102	49	-	-		
Average family Income (Scale 1-5)	Jews	lower	282	47			86.43^***^	0.82
		Average	119	20	2.75	1.60		
		Higher	109	18				
		Refuse to answer	90	15				
	Arabs	lower	167	80	1.62	1.08		
		Average	24	11				
		Higher	8	4				
		Refuse to answer	9	4				
Political attitudes(Scale 1-5)	Jews	Left	55	9	3.59	0.84	365.43^***^	0.83
		Center	203	34				
		Right	342	57				
	Arabs	Left	117	56	2.31	0.82		
		Center	82	39				
		Right	9	4				
Educational level(Scale 1-5)	Jews	High School	68	11	4.10	1.25	1.65	0.10
		Secondary	138	23				
		Academia	315	53				
	Arabs	High School	25	12	4.23	1.29		
		Secondary	41	20				
		Academia	112	54				

*** *p* < 0.001.

### Measures

#### Perceived partial social integration (PPSI)

Perceived partial social integration (PPSI) (in the context of the COVID-19 vaccination) is determined by a six-item scale about the COVID-19 pandemic (11). This scale is based on the assumption that the major issues that concern individuals who regard themselves as only partly socially integrated, are bothered by issues of social status, being appreciated by members of the mainstream of their society and limiting their free will (Example item: “Providing my full rights should precede any vaccination demand”). The response scale ranged from 1= Not true at all, to 5 = Very much true. The reliability of the PPSI scale in the Jewish sample was good (α = 0.79) and in the Arab sample was fair (α = 0.69).

#### Distress symptoms

The BSI scale (59) was employed. The present study included four items about anxiety and five items on depression. The response scale to this questionnaire ranges from 1 = not at all to 5 = to a very large extent. Respondents were asked to report the extent to which they are currently suffering from any of the problems presented. The internal reliabilities of this scale are very high: in both the Jewish sample (α = 0.89) and the Arab sample (α = 0.92).

#### Sense of danger

The present version included four items of the original sense of danger scale (22) with a reference to the COVID-19 pandemic. For example: “To what extent do you feel your life is in danger due to the coronavirus?” The response scale of the sense of danger index ranges from 1 = not at all, to 5 = to a very large extent. Good reliabilities were found for this scale among the Jewish sample (α = 0.81) and the Arab sample (α = 0.83).

#### Individual resilience (IR)

The original IR scale included 10 items [Connor and Davidson (60), cited by Campbell-Sills and Stein (61)]. In the present study, we used an abbreviated version of the Connor-Davidson questionnaire that includes two items. The response scale ranged from 0 = not true at all, to 4 = true almost all the time. Before the statistical analysis, we changed the scale to 1–5. The reliability of the abbreviated scale is fair in the Jewish sample (α = 0.66) and good in the Arab sample (α = 0.73).

#### Societal (national) resilience

This scale (29) reflects the major components of SR: patriotism, optimism, social integration, and trust in political and public institutions. This index has received much research support, both in Israel (40) and in additional countries (28). The abbreviated version employed in the current study includes 5 items. Example: “I have full confidence that the Israeli government makes the appropriate decisions in managing the COVID-19 crisis”. The response scale for the SR items ranges from 1 = strongly disagree to 6 = strongly agree. The reliability of the scale in the Jewish sample and the Arab sample were high: (α = 0.82) and (α = 0.90) respectively.

#### Optimism

A single item measured the level of optimism: “In light of the ongoing corona crisis, how would you define tour degree of optimism these days?” The response scale for this item ranges between 1 = very low to 5 = very high.

#### Level of vaccine uptake

Israeli residents were requested by February 2022, to be vaccinated at least three times against COVID-19. Specific vulnerable populations were called to be vaccinated with an additional (fourth) booster vaccine. The degree of vaccine uptake was determined by a single item: “To what extent are you currently vaccinated against the COVID-19?” The five-point response scale ranges from 1 = not vaccinated, to 5 = Vaccinated four times.

### Demographic variables

#### The income level

The income level was established by the following item: The average income of an Israeli family today is 18,671 NIS per month. Your family's income is 1. Much lower than this average; 2. Lower than this average; 3. Around this average; 4. Higher than this average; 5. Much higher than this average.

#### Education level

Education level was determined by the item “What is your education level?” The five response options were: 1. Primary education, 2. Secondary education, 3. Higher than secondary education (vocational), 4. Bachelor's degree, 5. Master's degree or higher.

## Results

Our first hypothesis claims that the Arab respondents will score higher on PPSI, distress symptoms and sense of danger, and will score lower on optimism, individual resilience, SR and vaccination uptake, compared with the Jewish respondents. The analysis of variance presented in Table 2 supports all these claims.

**Table 2:** Analysis of variance (ANOVA) comparing the psychological responses of Israeli Jewish (*n* = 600) and Arab (*n* = 208) respondents.

**Variable**	**Sample**	**M**	**SD**	**F**	***p***	**Cohen's d**
Vaccine level (Scale 1–5)	Jewish	3.68	1.00	−30.60	0.000	d = 0.38
	Arab	3.30	0.99			
Optimism (Scale 1–5)	Jewish	3.29	0.91	−5.27	0.022	d = 0.18
	Arab	3.12	0.96			
Societal resilience (Scale 1–6)	Jewish	3.58	0.77	−11.42	0.001	d = 0.25
	Arab	3.35	1.01			
Individual resilience (Scale 1–5)	Jewish	3.68	0.78	−16.17	0.000	d = 0.31
	Arab	3.42	0.91			
Anxiety symptoms (Scale 1–5)	Jewish	3.37	0.87	14.42	0.000	d = 0.30
	Arab	3.64	0.93			
Depressive symptoms (Scale 1–5)	Jewish	2.09	0.91	27.21	0.000	d = 0.41
	Arab	2.47	0.94			
Sense of danger (Scale 1–5) PPSI (Scale 1-5)	Jewish Arab Jewish	2.11 2.62 2.76	0.91 0.92 0.90	−48.63 −53.76	0.000 0.000	d = 0.56 d = 0.59
	Arab	3.27	0.71			

**Figure 1:**
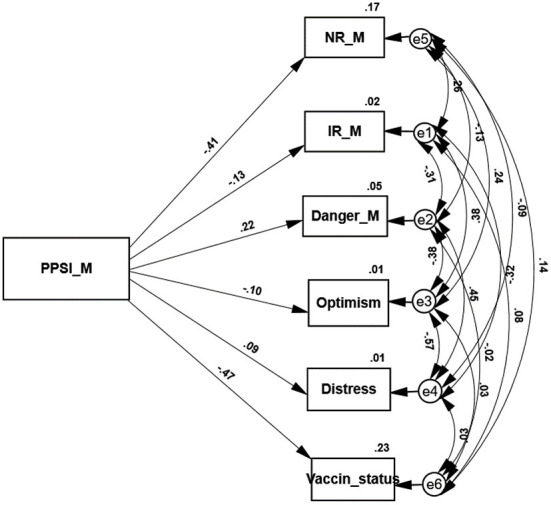
A path analysis of the variables predicted by the PPSI score of the Jewish sample.

**Figure 2:**
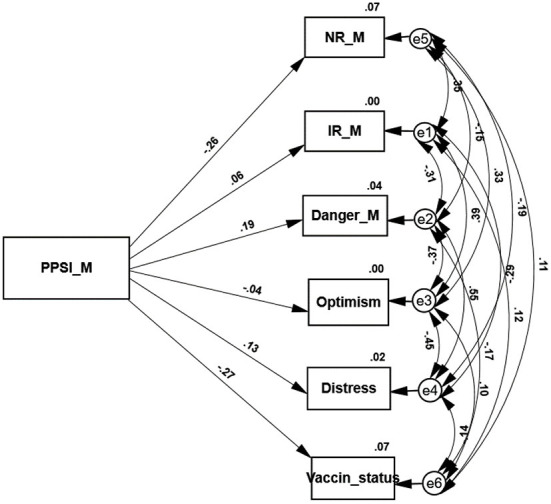
A path analysis of the variables predicted by the PPSI score of the Arab sample.

Two path analyses examine the variables that are predicted by the PPSI score of each sample (see Figures 1, 2). In agreement with hypothesis 2 higher PPSI scores of both groups significantly and negatively predict both SR and the level of vaccine uptake. Jewish as well as Arab individuals who regard themselves as only partly integrated into the Israeli society, have little trust in the national authorities and express a higher level of vaccine rejection. These path analyses support our third hypothesis as well. As expected, the path analysis conducted on the responses of the Jewish sample shows that their PPSI score positively predicts distress symptoms and negatively predicts indices of positive coping (IR and optimism). The second path analysis indicates that in agreement with hypothesis 3 the PPSI scores of the Arab sample do not significantly predict their level of distress symptoms or their resilience and optimism. The only exception is the sense of danger that is negatively predicted by their PPSI level.

## Discussion

The present study examines the role of perceived partial social integration (PPSI) in determining the level of rejection of the COVID-19 virus rejection and substantiates the claim that PPSI influences this behavior of members of two investigated ethno-cultural communities. A higher sense of only partial social integration was found to contribute to higher levels of vaccine rejection among members each of the two investigated groups. Arab as well as Jewish individuals rate themselves on the PPSI (COVID-19) scale, indicating that members of both groups are well aware of their relative social standing in the general society. As expected the Arab respondents regard themselves as significantly less integrated into the Israeli society than their Jewish counterparts. These results substantiate the generality of the PPSI concept and its suitability for assessing the perceived social conditions of different populations. Our earlier studies (11, 16) show that belonging to groups that are only partly integrated into their society is associated with a lower level of vaccination and a higher level of distrust in the state's authorities compared with the general population. According to Ali-Selah et al. (62), factors that may have contributed to the low levels of trust and adherence to be vaccinated among the Arab population include continued neglect by the healthcare system, disregard of the need to launch designated vaccination campaigns in Arabic, disregard of their particular needs and expectations, and their low socio-economic status. These varied elements strengthen the perceived exclusion of the Arab citizens from basic services. Gorelik et al. (63) that compared the vaccination rates among both Jewish and Arab citizens of Israel, found that the lowest compliance was noted concerning the Ultra-Orthodox group, a sector that is perceived as secluded from other groups in the Israeli society. The findings that higher PPSI scores are associated with a lower trust in the national authorities (i.e., societal resilience), in both ethnicities, indicates the important role of this concept in determining social perception.

Beyond the higher PPSI scores of the Arab respondents, our results point as well at different associations of PPSI with the personality attributes of the Jewish and the Arab samples. As hypothesized the members of the Arab minority report significantly higher levels of distress symptoms and a sense of danger, and significantly lower levels of individual resilience and optimism, compared with members of the Jewish majority. These results corroborate previous data on the relatively poor psychological status of the Arab minority in Israel (47).

However, despite the greater distress expressed by the Arab sample, the Jewish sample responds more harshly to the oppressive sense of PPSI. Our previous study (11) emphasized the role of PPSI in determining one's feelings and behavior in the process of coping with this pandemic. As expected, the PPSI of the Jewish sample associated with high levels of distress and sense of danger as well as by low levels of optimism, individual and social resilience. In contrast to this connection the Arab respondents dissociated their sense of being only partly integrated socially from these expressions of distress, exhibiting a kind of partial denial. Analyses of denial responses distinguish between a complete denial which repudiates, for example, a cancer diagnosis, and partial denial where the diagnosis of the disease is recognized, but neither its future prognostic implications nor the affective burden on the person are acknowledged (64, 65). Livneh (53) argues that partial denial is quite common in cases of serious illnesses. We conjecture that similar partial denial is prevalent as well in other domains, such as terrorism. The most important aspect of terrorism is the anxiety spread by it. Rather than totally denying this anxiety, the threatened people tend to fight it by employing partial denial, which enables them to dissociate this harsh risk from its impact on their future life (15).

Jewish as well as Arab citizens of Israel are well aware of their country's long history of wars and terror attacks that may recur as long as the Israeli-Arab conflict is not resolved (66). However, in a similar process of partial denial they rate a high-risk situation as lower than less risky threats (51). Kimhi et al. (67) demonstrate that the people of Israel regard the COVID-19 and their current economic threats persistently as more dangerous than terror attacks and acts of war. Furthermore, nuclear weapons, pandemics and global warning threaten to annihilate our species. Although we are unable to ignore these perils it seems that most people employ partial denial which helps them to keep these threats most of the time in the back of their minds, and live their lives without being constantly disturbed by them and by a host of other potential hazards.

We have assumed, therefore, that although the Arab respondents are aware of their low level of social integration and their high level of psychological distress, they manage to dissociate this distressing condition from its negative emotional outcomes. The path analysis conducted on their responses supports this analysis. Dissociation of the level of PPSI from the level of distress occurs among the Arab but not among the Jewish sample. We believe that the only exception to this rule, the significant association between the PPSI score of the Arab sample and the sense of danger, supports this analysis. The incessant discussion in the media of the pandemic risks did not give the Arab participants a chance to ignore or deny it.

One contribution of the present study to the research on social integration is presenting the role of PPSI in determining the behavior of vaccine rejection under stressful conditions of the COVID-19 pandemic. The role of PPSI and of its perception by members of a specific society in determining actual behavior have hardly been investigated in the integration literature (16, 41). The generality of the concept of PPSI and its consistent impact on the investigated behavior was supported by its similar impact on members of the Jewish majority and the Arab minority in Israel. We believe that the implications of a sense of PPSI of disadvantaged groups will be found in any social community or society and will contribute to a better understanding of the social processes, such as political inclination, that determine the interaction of these groups with the more established members of the investigated society.

## Limitations

The major limitation of the present study is the sampling which is based on an internet survey. In this method there is no way to determine the response rate since the survey continues until the determined number of respondents is reached and this may bias the sampling. However, experience shows that other methods of sampling fail to reach more representative large samples than internet panels.

## Conclusions

The present study replicates the finding of associating vaccine rejection levels with distrust in the intentions and the goodwill of the state's authorities (68). Health authorities tend to employ rational explanations to increase compliance of the general public with the requested precaution measures and with accepting the vaccine. The present study indicates that increasing the vaccination rates depend more substantially on trust in the authorities rather than on leveraging greater pressure on individuals that reject the vaccine. Previous research has found, in agreement with the present findings, that those who are uncertain about their social position and do not feel as an integral part of their society, are more prone to believe in conspiracy theories about being vaccinated, and distrust the authorities (16). Increased trust in the authorities and regarding oneself as an integral component of society are two vital conditions for vaccine acquiescence.

The level of distress of minority as well as majority groups in Israel reflects concurrently external as well as internal stressors. The external stressors may include economic and security threats, and in the case of the minority, different forms of discrimination. The internal stressors may consist of concerns about the present cohesiveness and the future of the community. Minority members may be further stressed by the conflicts raised by the process of modernization that requires changes of well-known old ways of living. The present study offers an additional internal stressor—the sense of PPSI that impacts the behavior of members of the majority as well as the minority. Previous research explains COVID-19 vaccine hesitancy and rejection by general lack of trust, objecting vaccines in general, or doubts about the efficiency of the vaccine (69). Eshel et al. (11) demonstrate that these explanations are more prevalent among vaccine refusing individuals who belong to several demographic categories. The present results show that a major reason for vaccine rejection is the stressful sense of insufficient social integration which is shared by the two investigated cultural groups.

Additional research is called for to better understand the concept of PPSI, and the often hidden conflict between the general society and some groups of its members, who are uncertain to what extent they belong socially. Furthermore, there is a need to understand whether and to what extent this experience of PPSI may change in line with changed social conditions, or whether it will be retained despite such changes. The answers to these questions are essential for any attempt to increase the public acceptance of vaccines and other measures aimed at curbing the present and future pandemics.

The heightened distress of the Arab society in Israel is generally explained by external reasons such as their lower political, social and economic status, the conflicts between them and the Jewish majority, or their being in a continuous process of modernization. An additional aspect of this distress is expressed by their sense of PPSI. The present Arab sample reports its high level of PPSI but dissociates it from the investigated indicators of distress, by means of partial denial of this association. Reducing the distress level of the Arab minority and improving their resilience level require a change of policy that is designed by the authorities. We believe that the best results will be obtained by policy measures that will improve the perceived integration of excluded groups into the Israeli society.

## Data availability statement

The analyzed raw data supporting the conclusions of this article will be made available by the authors, without undue reservation.

## Ethics statement

The studies involving human participants were reviewed and approved by Tel Aviv University Ethics Committee. The patients/participants provided their written informed consent to participate in this study.

## Author contributions

YE designed the study and drafted the first version of the manuscript. SK and YE analyzed the data. BA and SK conceptualized the full research. HM reviewed and revised the initial version. All authors read, revised, and contributed to the varied versions of the manuscript and approved the final version.

## Conflict of interest

The authors declare that the research was conducted in the absence of any commercial or financial relationships that could be construed as a potential conflict of interest.

## Publisher's note

All claims expressed in this article are solely those of the authors and do not necessarily represent those of their affiliated organizations, or those of the publisher, the editors and the reviewers. Any product that may be evaluated in this article, or claim that may be made by its manufacturer, is not guaranteed or endorsed by the publisher.
